# Supplementary material for: Development and validation of a multi-dimensional diagnosis-based comorbidity index that improves prediction of death in men with prostate cancer: Nationwide, population-based register study
Source: PLoS One. 2024 Jan 18;19(1):e0296804. doi: 10.1371/journal.pone.0296804 (PMC10796041; doi:10.1371/journal.pone.0296804)
Supplement: S2 Appendix — (DOCX) [file pone.0296804.s002.docx]

# Appendix S2: Supplementary Methods.

## 0. Content

In the following, we describe how we extracted, cleaned, and processed the ICD-10 codes and created the predictors**.** We also provide additional details on how the variable selection and model fitting was performed.

## 1. ICD-10 code extraction and cleaning

This was performed in four steps and the procedure is summarized in **S1 Figure.**

*Step 0. Code extraction and cleaning.* All registrations of hospital discharges up to 10 years prior to the index date of each man were extracted from the National Patient Register. Each registration consisted of one ICD-10 code along with a date for admission to hospital (only for registrations in the Inpatient register) and a date for discharge. Codes were cleaned by removing special characters (e.g. punctuations). Registrations with an erroneous code (not recognized as an ICD-10 code) were subsequently discarded. The Swedish version of ICD-10 (ICD-10-SE) permits 3-5 characters and may end with either a letter or digit. A code was considered erroneous if it consisted of only digits or had less than three or more than five characters. Duplicate registrations, and registrations with a code indicating prostate cancer (C61 and C619) were also discarded.

A total of 10 325 unique codes were extracted.

## 2. Code truncation, elongation, pruning, and filtering

Codes may be registered in the National Patient register using a varying number of characters, e.g. I73 (other peripheral vascular diseases) and I731 (thromboangiitis obliterans). To handle this variation in coding, we processed the codes as described below.

*Step 1: Truncation and elongation*. For each *N*=2, 3, 4 and 5, all registered codes were first truncated to contain at most *N* characters. Any observed ICD-10 codes that were shorter than *N* were elongated by adding 9 (indicating subcategory *other* or *unspecified*) at the end until it reached *N* number of characters.

*Step 2: pruning of unnecessary codes*. In step 1, some codes may be created using more characters than needed which may introduce identical predictors using different number of characters. At a given number of character *N,* we only kept a code that contributed with additional information in the last position relative to the same code truncated to *N-1* characters*.*

*Step 3: code filtering.* Codes had to be present in at least 0.01% of the development cohort to be included in the development of the multidimensional diagnosis-based comorbidity index.

A full list of all processed ICD-10 codes that generated the predictors can be provided upon request from the corresponding author**.**

## 3. An example

We want to create predictors using *N=*4 number of characters and find that both codes I731 and I73 are registered in the National Patient Register. I73 is first elongated to I739 (peripheral vascular disease, unspecified), as described in step 1. Both codes (I731 and I739) are then used to generate the predictors.

If, on the other hand, there are no four-character codes registered starting with I73, except possibly I739; then I739 is disregarded when generating predictors with *N=*4, as described in step 2, since it will produce the exact same predictors as the (truncated) code I73 at *N=*3 characters.

Note that in this example, I73, I731 and I739 may still be contributing to the predictors generated at *N=*2 (all truncated to I7) and *N=*3 (all truncated to I73).

## 4. Definition of predictors

Four different variants of each registered ICD-10 code could contribute meaningful information, depending on the number of characters used. For example, the code I731 (thromboangiitis obliterans) could potentially contribute information based on two (I7), three (I73), four (I731), and/or five characters (I7319). Each of the four variants of the registered ICD-10 code was then the basis for code-specific indicator variables (taking values yes/no) that for each ICD-10 code accounted for occurrence, frequency, recency, and total duration of hospitalization in the 10 years preceding the index date. There were 12 351 processed codes and 5612 of these were present in at least 0.01% of the development cohort.

The predictors consisted of the 10 dummy variables described below, and these were created separately for each of the 5612 codes **(S1 Figure)**.

**Occurrence:** ICD-10 code registered

- as primary diagnosis
- as primary or secondary diagnosis

**Frequency:** ICD-10 code registered as primary diagnosis

- on at least 2 unique dates
- on at least 3 unique dates
- on at least 4 unique dates

**Recency:** ICD-10 code registered as primary diagnosis

- within 90 days prior to index date
- within 180 days prior to index date
- within 365 days prior to index date

**Duration:** total number of days in hospital summed over all hospitalizations with the respective ICD-10 code at unique dates as primary diagnosis

- at least 7 days
- at least 14 days

The total duration of a hospital admission associated with an ICD-10 code was defined as the difference between the registered dates for hospital admission and discharge respectively in the inpatient register. Durations ≤ 0 were set to 0.5 days, and for outpatient visits the duration of stay was also set to 0.5 days.

## 5. Remark on how to compute the multidimensional diagnosis-based comorbidity index

To compute the multidimensional diagnosis-based comorbidity index given a set of coefficients it is only required to perform steps 0 and 1 above. After that, one needs to multiply the predictors with the corresponding coefficients and sum all the products to obtain the MDCI for one individual.

## 6. Variable selection and model fitting

The analysis was performed using R version 4.1.3 [1]. We used a Cox proportional hazards regression model with all the predictors as covariates and with age-stratified baseline hazard (age cutoffs at ≤50, 51-60, 61-65, 71-75, 81-85, 86-90, ≥91) [2, 3]. We applied regularization by use of the elastic net, using the R-package *glmnet* version 4.1-2 [4, 5], to simultaneously perform both variable selection and handle collinearity. Elastic net combines the least absolute shrinkage and selection operator (LASSO) and Ridge penalties by imposing a penalty hyper-parameter λ and a hyper-parameter α that balances the two penalties. If $\beta_{j}$ is the j^th^ model parameter, the complete penalty term added to the negative log-likelihood is:

$\lambda\left[ \left( 1-\alpha\right)\sum_{j} \beta_{j}^{2}/2+ \alpha\sum_{j} \left| \beta\right|_{j} \right]$.

We set α = 0.5 to balance the LASSO and Ridge penalties equally.

Ten-fold cross-validation was used to estimate the concordance index (c-index) for each model over a grid of 100 values of λ [6, 7]. A c-index close to 0.5 indicates poor discriminatory accuracy while a c-index closer to 1 indicates good discriminatory accuracy. The λ with the largest average c-index obtained from the cross-validation procedure was selected. Within each of the 10 cross-validation folds, constant predictors were removed before parameter estimation and hyper-parameter selection using the *filter* argument to *cv.glmnet*. The selected penalty hyper-parameter from the cross-validation and associated c-indices can be found in **S1 Table.**

All included predictors along with the corresponding coefficients for the MDCI developed using 10-years of follow-up for mortality can be found in **S2 Table**.

## 7. Predictive performance

The final model coefficients were obtained by fitting the model to the entire development cohort using the optimal hyperparameter obtained from the cross-validation procedure. The MDCI was subsequently computed for each man in all three cohorts by computing the linear predictor. The discrimination of the MDCI was measured by the c-index, and calibration was assessed by calibration curves [8]. The calibration curve was obtained by comparing predicted and observed survival at the end of follow-up in strata defined by deciles of the MDCI based on the entire development cohort. The observed survival probability was the Kaplan-Meier estimate at the end of follow-up in each stratum. The predicted survival probability was obtained by using the MDCI as a predictor in a Cox proportional hazards model and extracting the estimated survival probability for each individual at the corresponding end of follow-up after estimating the baseline hazard function [9]. These predicted survival probabilities were subsequently averaged within each stratum.

## References

[1] R Core Team. R: A language and environment for statistical computing. R Foundation for Statistical Computing, Vienna, Austria. 2022.

[2] Cox DR. Regression models and life‐tables. Journal of the Royal Statistical Society: Series B (Methodological). 1972;34:187-202.

[3] Gedeborg R, Sund M, Lambe M, Plym A, Fredriksson I, Syrjä J, et al. An aggregated comorbidity measure based on history of filled drug prescriptions: development and evaluation in two separate cohorts. Epidemiology. 2021;32:607-15.

[4] Simon N, Friedman J, Hastie T, Tibshirani R. Regularization paths for Cox’s proportional hazards model via coordinate descent. Journal of statistical software. 2011;39:1.

[5] Friedman J, Hastie T, Tibshirani R. Regularization paths for generalized linear models via coordinate descent. Journal of statistical software. 2010;33:1.

[6] Hastie T, Tibshirani R, Friedman JH, Friedman JH. The elements of statistical learning: data mining, inference, and prediction: Springer; 2009.

[7] Stone M. Cross‐validatory choice and assessment of statistical predictions. Journal of the royal statistical society: Series B (Methodological). 1974;36:111-33.

[8] Harrell F. Regression modeling strategies: Springer Science+Business Media, Inc.; 2001.

[9] Therneau T. A package for survival analysis in S. R package version. 2015;2.
