# Supplementary material for: Development and validation of a multi-dimensional diagnosis-based comorbidity index that improves prediction of death in men with prostate cancer: Nationwide, population-based register study
Source: PLoS One. 2024 Jan 18;19(1):e0296804. doi: 10.1371/journal.pone.0296804 (PMC10796041; doi:10.1371/journal.pone.0296804)
Supplement: S1 Fig — (PDF) [file pone.0296804.s007.pdf]

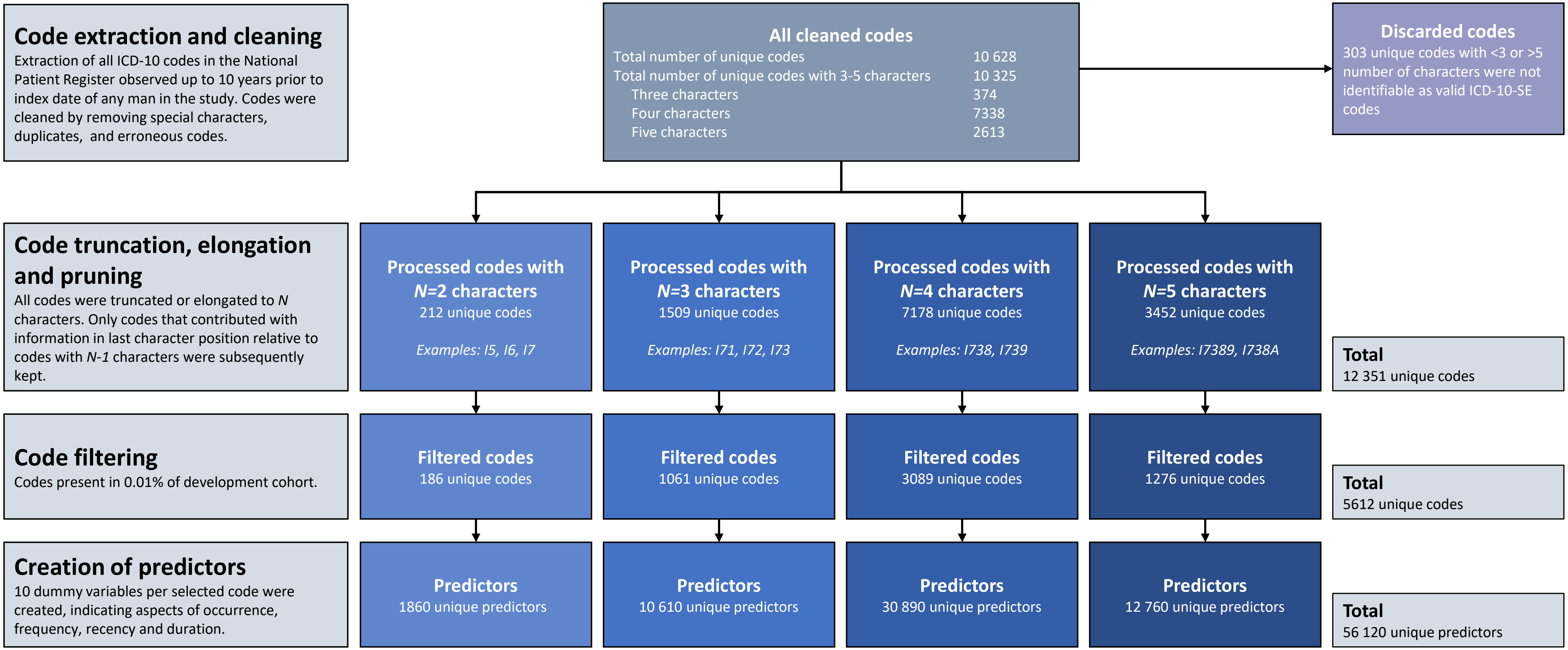
Code extraction and cleaning
Extraction of all ICD-10 codes in the National Patient Register observed up to 10 years prior to index date of any man in the study. Codes were cleaned by removing special characters, duplicates, and erroneous codes.
All cleaned codes
Total number of unique codes 10 628
Total number of unique codes with 3-5 characters 10 325
Three characters 374
Four characters 7338
Five characters 2613
Discarded codes
303 unique codes with <3 or >5 number of characters were not identifiable as valid ICD-10-SE codes
Code truncation, elongation and pruning
All codes were truncated or elongated to N characters. Only codes that contributed with information in last character position relative to codes with N-1 characters were subsequently kept.
Processed codes with N=2 characters
212 unique codes
Examples: I5, I6, I7
Processed codes with N=3 characters
1509 unique codes
Examples: I71, I72, I73
Processed codes with N=4 characters
7178 unique codes
Examples: I738, I739
Processed codes with N=5 characters
3452 unique codes
Examples: I7389, I738A
Total
12 351 unique codes
Code filtering
Codes present in 0.01% of development cohort.
Filtered codes
186 unique codes
Filtered codes
1061 unique codes
Filtered codes
3089 unique codes
Filtered codes
1276 unique codes
Total
5612 unique codes
Creation of predictors
10 dummy variables per selected code were created, indicating aspects of occurrence, frequency, recency and duration.
Predictors
1860 unique predictors
Predictors
10 610 unique predictors
Predictors
30 890 unique predictors
Predictors
12 760 unique predictors
Total
56 120 unique predictors
