# Supplementary material for: Development and validation of a multi-dimensional diagnosis-based comorbidity index that improves prediction of death in men with prostate cancer: Nationwide, population-based register study
Source: PLoS One. 2024 Jan 18;19(1):e0296804. doi: 10.1371/journal.pone.0296804 (PMC10796041; doi:10.1371/journal.pone.0296804)
Supplement: S2 Fig — (PDF) [file pone.0296804.s008.pdf]

All predictors

Extracted from the processed codes.

Predictors with *N*=2 characters	Predictors with *N*=3 characters	Predictors with *N*=4 characters	Predictors with *N*=5 characters
186 unique codes<br>1860 unique prognostic factors	1061 unique codes<br>10610 unique prognostic factors	3089 unique codes<br>30890 unique prognostic factors	1276 unique codes<br>12760 unique prognostic factors

Selected codes and predictors

Predictors selected in the final multidimensional diagnosis-based comorbidity index (MDCI) after cross-validation.

	N=2: MDCI developed using 1 year of follow-up	*N*=2: MDCI developed using 5 years of follow-up	*N*=2: MDCI developed using 10 years of follow-up	*N*=3: MDCI developed using 1 year of follow-up	*N*=3: MDCI developed using 5 years of follow-up	*N*=3: MDCI developed using 10 years of follow-up	*N*=4: MDCI developed using 1 year of follow-up	*N*=4: MDCI developed using 5 years of follow-up	*N*=4: MDCI developed using 10 years of follow-up	*N*=5: MDCI developed using 1 year of follow-up	*N*=5: MDCI developed using 5 years of follow-up	*N*=5: MDCI developed using 10 years of follow-up
Unique codes	73	114	123	114	267	291	134	344	390	48	160	174
Predictors	154	285	311	161	411	462	170	465	530	62	217	240
Occurrence	49 (32%)	96 (34%)	102 (33%)	83 (52%)	218 (53%)	261 (56%)	109 (64%)	316 (68%)	362 (68%)	36 (58%)	130 (60%)	145 (80%)
Frequency	16 (10%)	49 (17%)	53 (17%)	17 (11%)	80 (19%)	84 (18%)	17 (10%)	67 (14%)	81 (15%)	8 (13%)	37 (17%)	43 (18%)
Recency	51 (33%)	81 (28%)	84 (27%)	42 (26%)	84 (20%)	87 (19%)	32 (19%)	56 (12%)	57 (11%)	13 (21%)	30 (14%)	36 (15%)
Duration	38 (25%)	59 (21%)	72 (23%)	19 (12%)	29 (7%)	30 (6%)	12 (7%)	26 (6%)	30 (6%)	5 (8%)	20 (9%)	16 (7%)

Summary of selected codes and predictors

	MDCI developed using 1 year of follow-up	MDCI developed using 5 years of follow-up	MDCI developed using 10 years of follow-up
Unique codes	369	885	978
Predictors			
Total	547	1378	1543
Occurrence	277 (51%)	760 (55%)	870 (56%)
Frequency	58 (11%)	233 (17%)	261 (17%)
Recency	138 (25%)	251 (18%)	264 (17%)
Duration	74 (14%)	134 (10%)	148 (10%)
