# Supplementary material for: Development and validation of a multi-dimensional diagnosis-based comorbidity index that improves prediction of death in men with prostate cancer: Nationwide, population-based register study
Source: PLoS One. 2024 Jan 18;19(1):e0296804. doi: 10.1371/journal.pone.0296804 (PMC10796041; doi:10.1371/journal.pone.0296804)
Supplement: S3 Fig — ICD-10 codes with 2–5 characters in each circle (inner circle = 2 characters, outer circle = 5 characters) and grouped predictors (occurrence, frequency, recency, duration). Each predictor for each code corresponds to a circle segment and this segment is colored if any coefficient within that group of predictors was included in the multi-dimensional diagnosis-based comorbidity index developed using 1, 5, and 10 years of follow-up for mortality, respectively. (PDF) [file pone.0296804.s009.pdf]

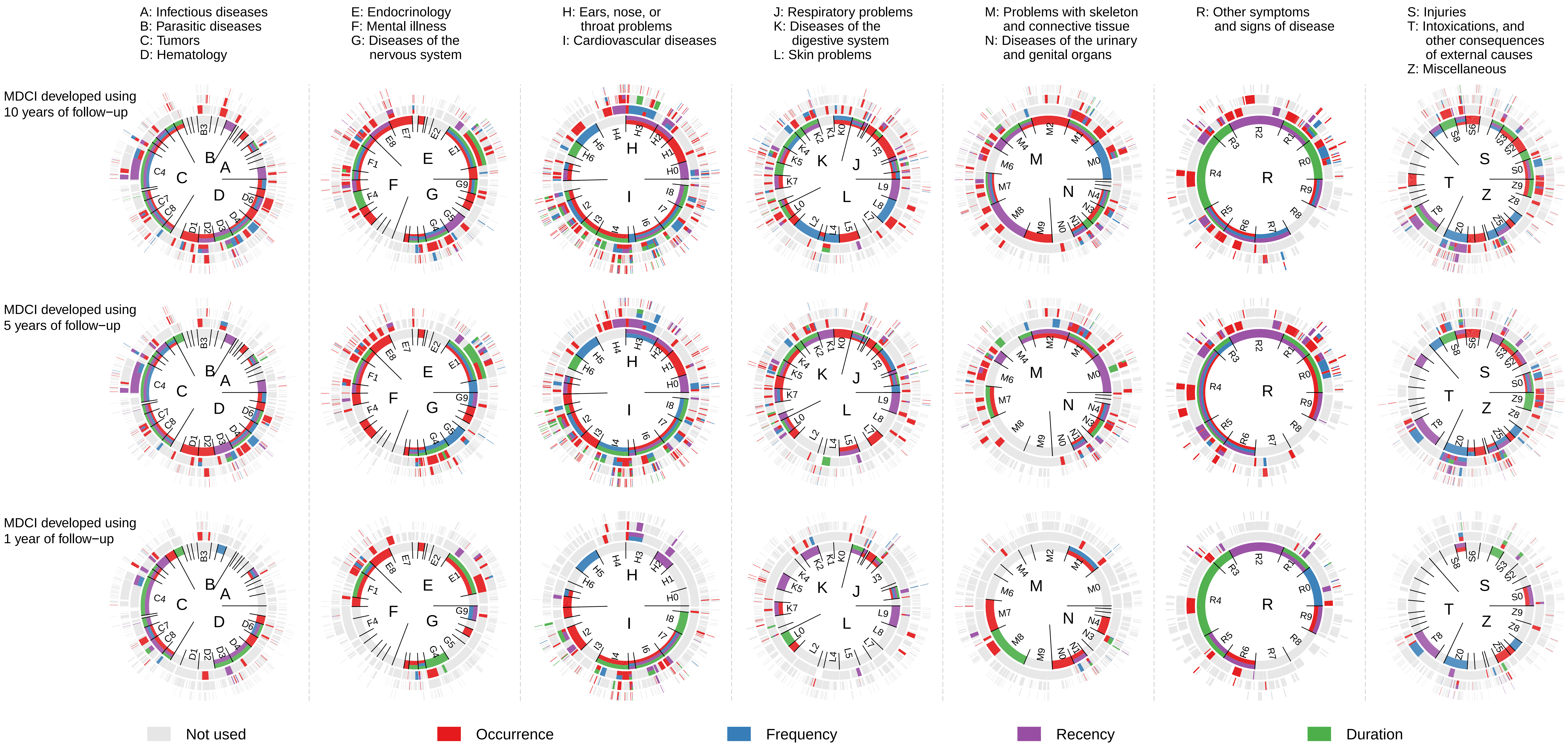

A: Infectious diseases
B: Parasitic diseases
C: Tumors
D: Hematology
E: Endocrinology
F: Mental illness
G: Diseases of the nervous system
H: Ears, nose, or throat problems
I: Cardiovascular diseases
J: Respiratory problems
K: Diseases of the digestive system
L: Skin problems
M: Problems with skeleton and connective tissue
N: Diseases of the urinary and genital organs
R: Other symptoms and signs of disease
S: Injuries
T: Intoxications, and other consequences of external causes
Z: Miscellaneous
MDCI developed using 10 years of follow−up
MDCI developed using 5 years of follow−up
MDCI developed using 1 year of follow−up
Not used
Occurrence
Frequency
Recency
Duration
