# Supplementary figures and images for: Development and validation of a multi-dimensional diagnosis-based comorbidity index that improves prediction of death in men with prostate cancer: Nationwide, population-based register study
Source: PLoS One. 2024 Jan 18;19(1):e0296804. doi: 10.1371/journal.pone.0296804 (PMC10796041; doi:10.1371/journal.pone.0296804)

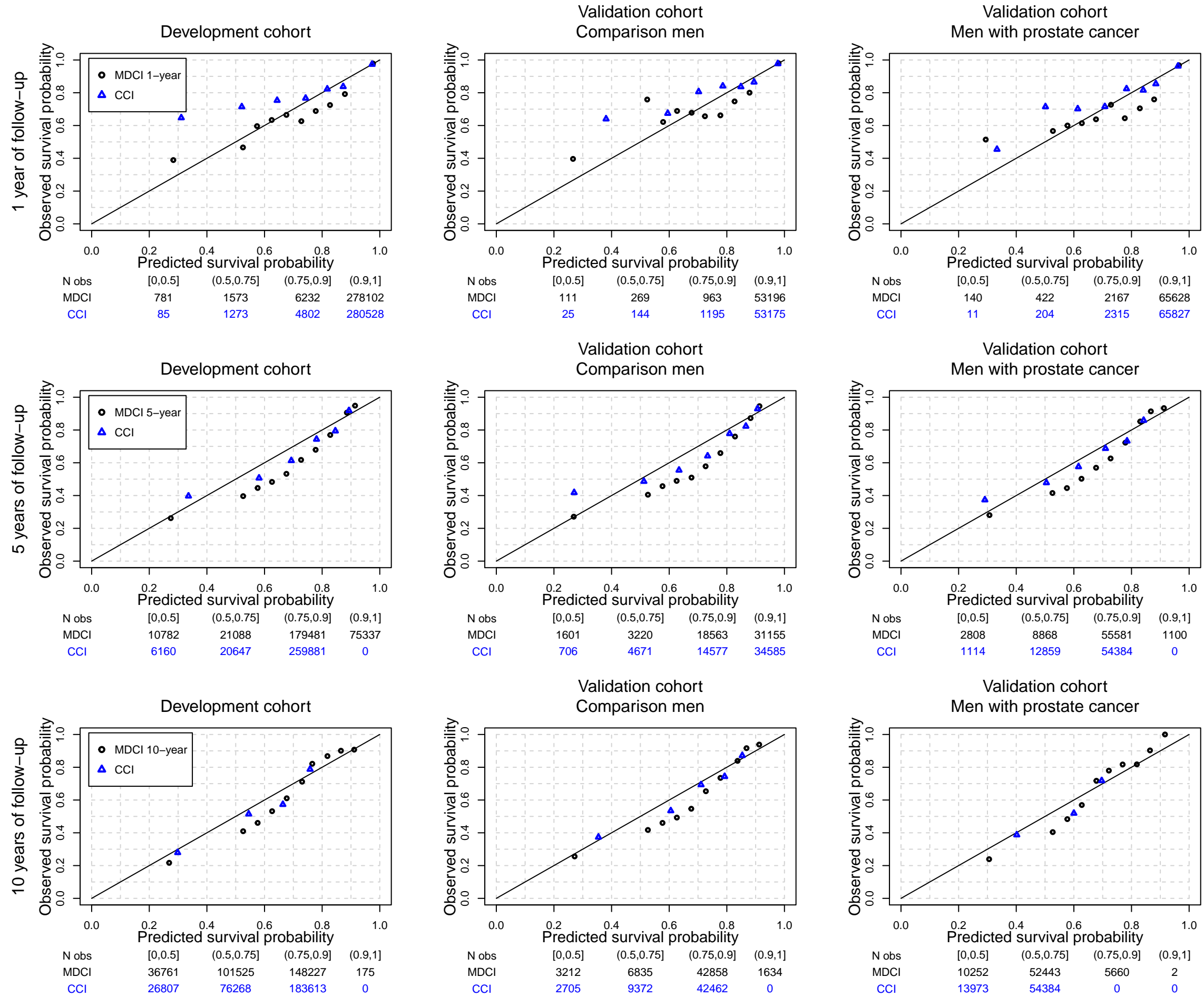

Supplement: S4 Fig — Observed 1, 5, and 10-year mortality risk compared to predicted risk based on the MDCI developed using 1, 5, and 10 years of follow-up for mortality, respectively, or the CCI. (PDF) [file pone.0296804.s010.pdf]

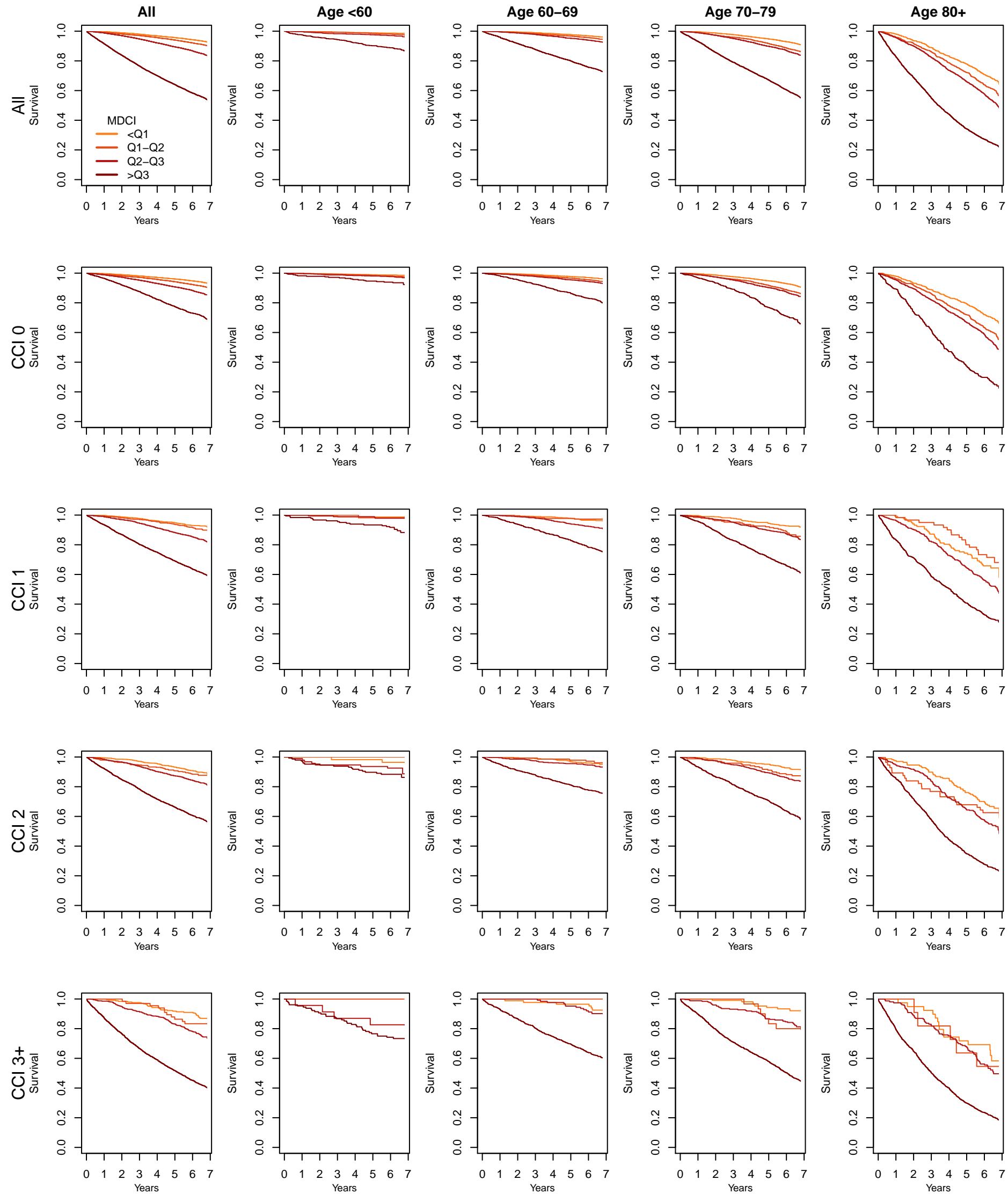

Supplement: S5 Fig — Developed using 10 years of follow-up for mortality vs the Charlson comorbidity index (CCI) in the validation cohort of comparison men. (PDF) [file pone.0296804.s011.pdf]

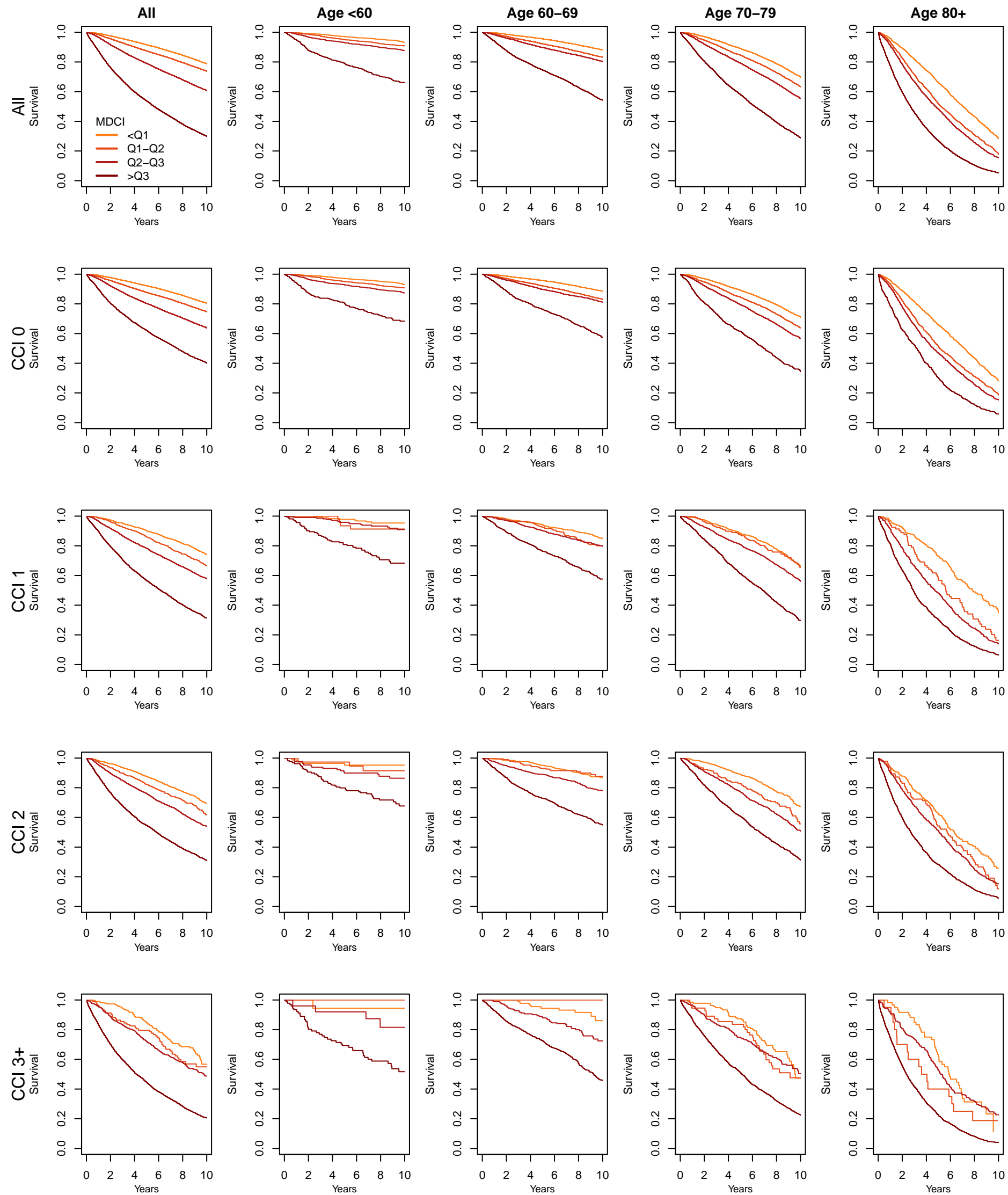

Supplement: S6 Fig — Developed using 10 years of follow-up for mortality vs the Charlson comorbidity index (CCI) in the validation cohort of men with prostate cancer. (PDF) [file pone.0296804.s012.pdf]

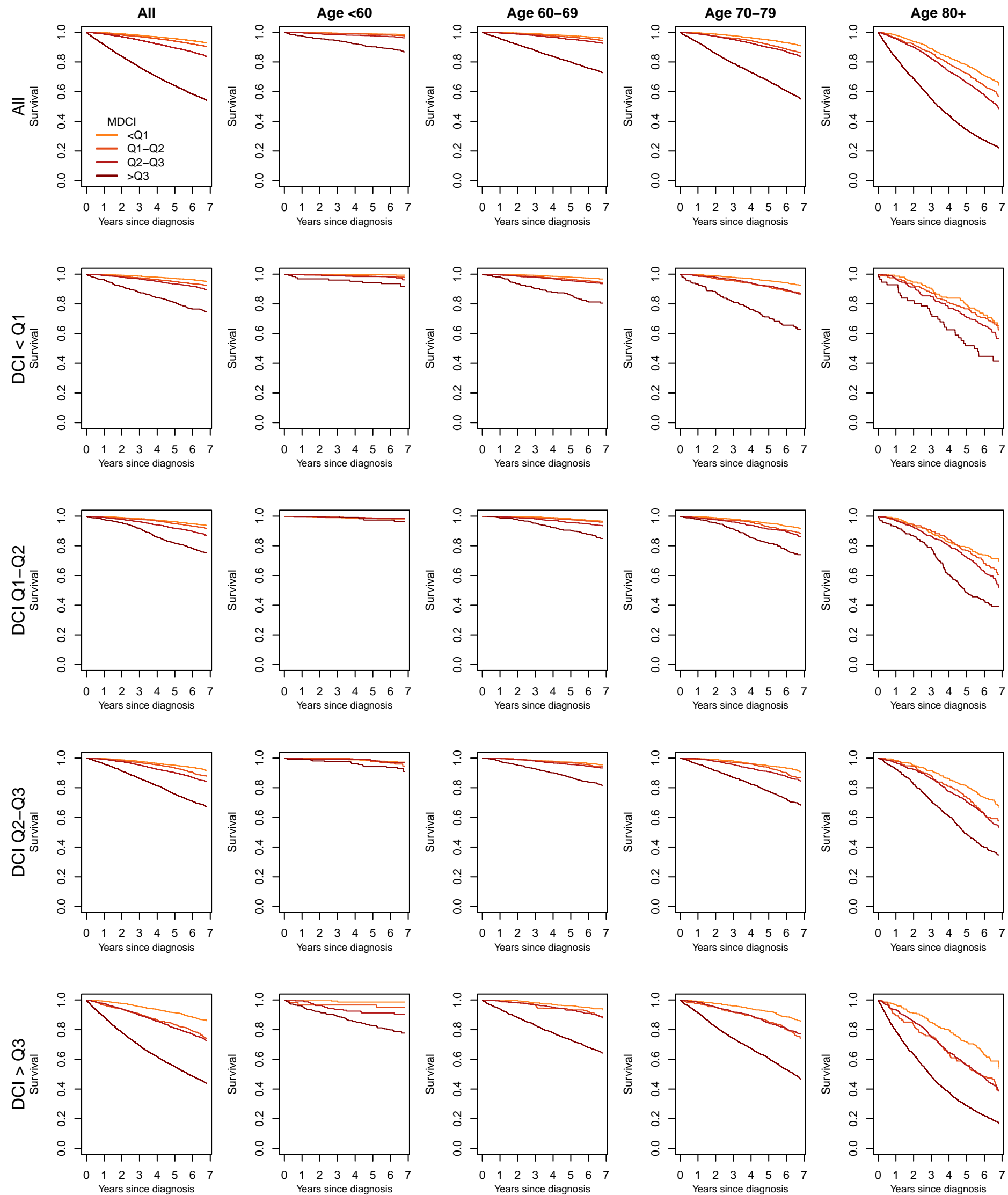

Supplement: S7 Fig — Developed using 10 years of follow-up for mortality vs the drug comorbidity index (DCI) in the validation cohort of comparison men. (PDF) [file pone.0296804.s013.pdf]

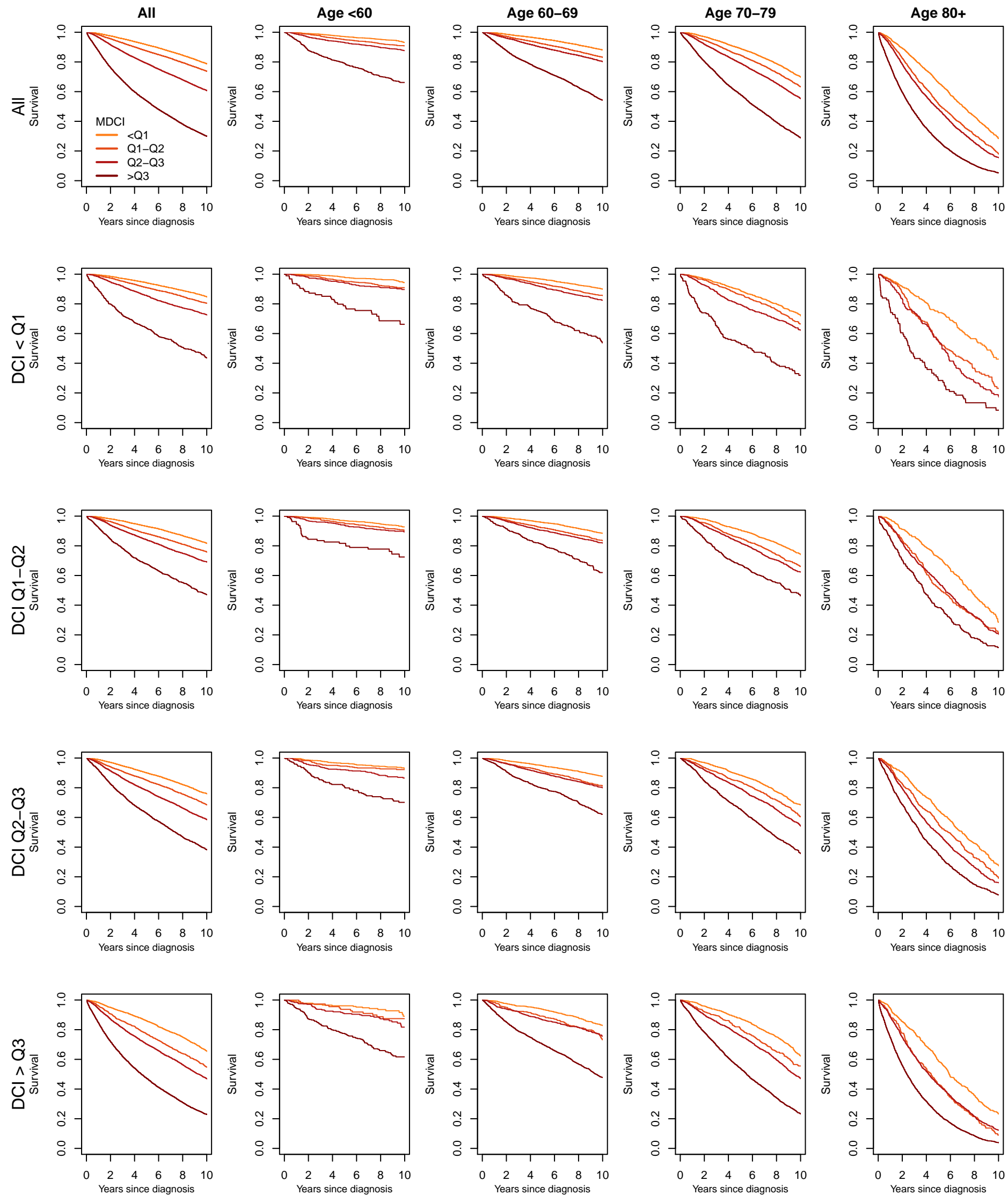

Supplement: S8 Fig — Developed using 10 years of follow-up for mortality vs the drug comorbidity index (DCI) in the validation cohort of men with prostate cancer. (PDF) [file pone.0296804.s014.pdf]
