# Supplementary material for: Development and validation of a multi-dimensional diagnosis-based comorbidity index that improves prediction of death in men with prostate cancer: Nationwide, population-based register study
Source: PLoS One. 2024 Jan 18;19(1):e0296804. doi: 10.1371/journal.pone.0296804 (PMC10796041; doi:10.1371/journal.pone.0296804)
Supplement: S9 Fig — Comparing men with a multi-dimensional diagnosis-based comorbidity index (MDCI) below Q1 vs men with MDCI above Q3. The analysis was also stratified by age, Charlson comorbidity index (CCI) and drug comorbidity index (DCI). NA indicates that the hazard ratio could not be estimated due to small sample size and/or few events in each stratum. (PDF) [file pone.0296804.s015.pdf]

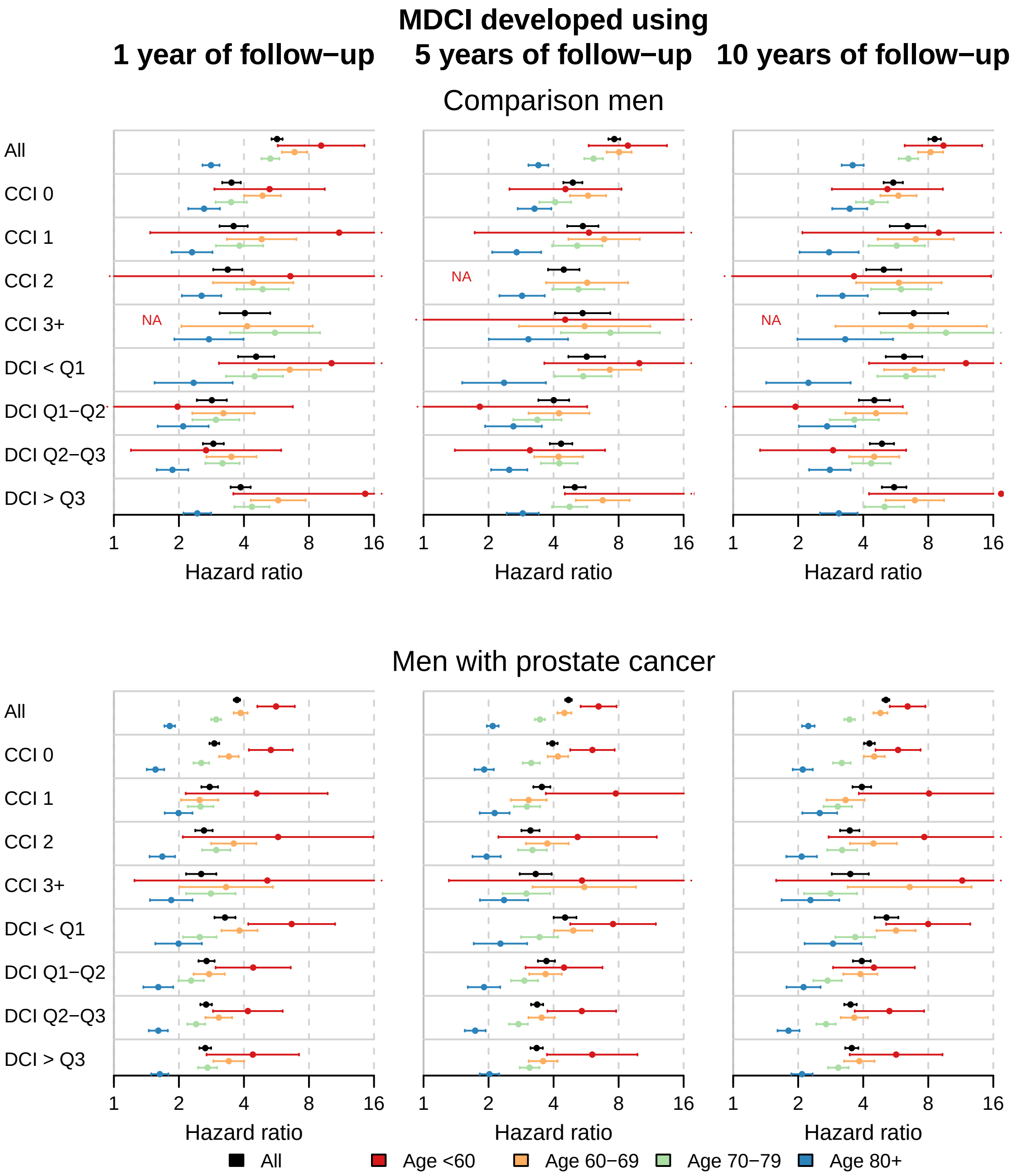
MDCI developed using
1 year of follow−up
5 years of follow−up
10 years of follow−up
Comparison men
Men with prostate cancer
All
CCI 0
CCI 1
CCI 2
CCI 3+
DCI < Q1
DCI Q1−Q2
DCI Q2−Q3
DCI > Q3
NA
1
2
4
8
16
Hazard ratio
All
Age <60
Age 60−69
Age 70−79
Age 80+
