# Supplementary material for: Development and validation of a multi-dimensional diagnosis-based comorbidity index that improves prediction of death in men with prostate cancer: Nationwide, population-based register study
Source: PLoS One. 2024 Jan 18;19(1):e0296804. doi: 10.1371/journal.pone.0296804 (PMC10796041; doi:10.1371/journal.pone.0296804)
Supplement: Attachment — Submitted filename: Answer_to_reviewers_20231204.docx [file pone.0296804.s017.docx]

Reviewer #1: The authors have done a very nice job responding to almost all of my comments. I appreciate the additional details and explanations. The revised manuscript and supplemental materials are substantially improved and nearly ready for publication. In my view only 4 things remain to be done.

We thank the Reviewer for the constructive comments that we have attempted to fully address below.

Most importantly, I respectfully disagree with the authors' response to my suggestion to disaggregate their index's performance to aid other scientists in understanding whether the improved predictive ability is due to a longer lookback, addition of medications, or addition of comorbidities. This is actually one of the key insights of this paper, even recognizing the limitations and challenges the authors provide. It is not difficult to do an illustrative analysis rather than a comprehensive analysis. For example, for the outcome of 10-year overall survival, show the performance of the CCI, then extend lookback, then add additional conditions, then add medications. Report the C-statistic for each step. No need for external validation.
**Answer:** We thank the reviewer for the suggestions and have made complementary analyses based on the development dataset in line with the suggestions.

We first assessed the impact of lookback on the CCI and/or in combination with the DCI (drug comorbidity index) (see table below). We found that a longer lookback increases the C-index, in particular when CCI is used alone, and that the C-index increases when CCI is combined with DCI, in which case lookback has some, but less, impact.

| **Lookback for CCI (years)** | **Variables** | **C-index** | **95% CI** |
| --- | --- | --- | --- |
| 1 | CCI | 0.599 | (0.597 - 0.601) |
| 1 | CCI and DCI | 0.715 | (0.713 - 0.717) |
| 5 | CCI | 0.654 | (0.652 - 0.655) |
| 5 | CCI and DCI | 0.721 | (0.719 - 0.723) |
| 10 | CCI | 0.668 | (0.666 - 0.670) |
| 10 | CCI and DCI | 0.724 | (0.722 - 0.725) |

Note that the DCI has a fixed lookback of 1 year.

We also similarly assessed the impact of lookback for the MDCI (see table below). The findings were comparable to the above although the C-indices were slightly larger overall. In particular, MDCI using a lookback of 10 years performed better than CCI and DCI combined, while MDCI and DCI combined performed the best.

| **Lookback for MDCI (years)** | **Variables** | **C-index** | **95% CI** |
| --- | --- | --- | --- |
| 1 | MDCI | 0.637 | (0.635 - 0.639) |
| 1 | MDCI and DCI | 0.722 | (0.720 - 0.724) |
| 5 | MDCI | 0.716 | (0.714 - 0.718) |
| 5 | MDCI and DCI | 0.743 | (0.742 - 0.745) |
| 10 | MDCI | 0.734 | (0.732 - 0.735) |
| 10 | MDCI and DCI | 0.751 | (0.749 - 0.752) |

Note that the DCI has a fixed lookback of 1 year.

The above findings have been summarized in a new Supplementary Table 3, and we have made the following comment in the methods section:

“In an exploratory analysis using the development dataset we also evaluated the impact of length of lookback period for both the Charlson comorbidity index and the MDCI, alone or in combination with the drug comorbidity index.”

We have made the following comments in the results section:

“In complementary analyses, decreasing the length of the lookback period to 5 years or 1 year for the Charlson comorbidity index or for the MDCI clearly decreased the predictive ability, e.g. using a lookback of one year the C-index for the Charlson comorbidity index and MDCI were 0.599	(95% CI: 0.597 - 0.601) and 0.637 (0.635 - 0.639), respectively (Supplementary Table S3). When the comorbidity indices were combined with the drug comorbidity index the overall predictive ability increased and the impact of lookback was less pronounced, although a lookback of 10 years produced the largest C-indices both for Charlson comorbidity index and the MDCI, 0.724 (95% CI: 0.722 - 0.725) and 0.751 (95% CI: 0.749 - 0.752), respectively (Supplementary Table S3).”

We have made the following changes in the discussion (new text in **bold**):

“Our results suggest that the MDCI and DCI reflect different aspects of a patient’s medical history and therefore should be used in combination**, preferably with a lookback of 5-10 years for the MDCI.”**

and

“While the study population was large, the results may be influenced by the restriction to men and the distribution of comorbidities in this particular population. The study population was therefore not well suited to elucidate the relative contribution of predictors based on occurrence, frequency, duration, and recency to the overall performance of the MDCI. The main purpose of this study was to provide proof of principle for a multidimensional diagnosis-based comorbidity index.”

Second, while I like revised Figure 1, it would be helpful to have English labels/diagnoses for each level of characters, e.g. I5, I51, I500, to allow readers to better understand the level of specificity being illustrated.

**Answer:** We thank the reviewer for the suggestion. We have improved the figure based on the suggestion and updated the figure text (see below).

Updated figure text **(new text in bold):**

**“**Figure 1. Example of predictors selected in the prediction model. ICD-10 codes for cardiovascular diseases are shown in the figure, specifically focusing on the ICD-10 code category I5 (heart failure and some heart disorders and diseases). The innermost circle represents ICD-10 codes with character length 2 and the outmost circle codes with character length 5 (inner circle=length 2, outer circle=length 5) and grouped predictors. Each predictor variable (occurrence, frequency, recency, duration) derived for each ICD-10 code corresponds to a color-coded circle segment. A segment is colored if any coefficient within that group of predictors had a non-zero coefficient for that code, and grey if non-informative. **Unspec. = unspecified subcategory.**”

Third, with at least 16 supplemental materials, I find this incredibly comprehensive but excessive. Some things can be combined or removed. For example, table S6 and S7 can be combined. Tables S1 and S4 could be removed in my view or made available upon writing to the authors.

**Answer:** We thank the reviewer for the suggestion and agree. We have removed Tables S1, S3 and S4 from the supplementary materials and combined tables S6 and S7. All removed tables are of course available upon request.

Finally, figure S4 would benefit from a blow-out of the predicted survival in the 0.8-1.0 or 0.9-1.0 range, where 'most of the action is'. This could either be done using a non-linear graph or a graph within graph, at least for the 3 development cohort figures (otherwise we have 9 graphs to revise).

**Answer:** We agree that the figure can be improved. We had actually revised the figure before but accidentally included the wrong version in the submission. The revised figure is based the same computations that underly Figure 2, and the points are now better spread out (see below).

Reviewer #2: The authors have made improvements to the figures/tables to improve the interpretability of their work. They provide reasonable explanation for the scope and aim of their work in response to some methodological critiques, especially in light of their ongoing follow-up studies within this domain.

**Answer:** We thank the reviewer for this feedback.

In conclusion we thank the Editor and the Reviewers for the suggestions that have led to further improvements of the manuscript and hope that it is now suitable to be considered for publication in PLOS ONE.

On behalf of the authors,

Marcus Westerberg, PhD

Department of Surgical Sciences,

Uppsala University

e-mail: marcus.westerberg@uu.se
